# Letters to the editor in response to studies of guns in the home and homicide and suicide

**DOI:** 10.1186/s40621-016-0100-9

**Published:** 2017-02-06

**Authors:** Douglas J. Wiebe, Kalen Flynn, Charles C. Branas

**Affiliations:** 1 Department of Biostatistics and Epidemiology, Perelman School of Medicine, University of Pennsylvania, 423 Guardian Drive, Blockley Hall Room 902, Philadelphia, PA 19103 USA; 2 School of Social Policy and Practice, University of Pennsylvania, Philadelphia, PA USA

**Keywords:** Firearm, Gun, Homicide, Suicide, Letters to the editor

## Abstract

**Background:**

Letters to the editor are an important venue for scientific discussion and ensuring accountability of authors and editors. We investigated the content and tone of letters to the editor published in response to research on having a firearm in the home as it relates to homicide and suicide.

**Methods:**

A recent meta-analysis found 16 analytic studies of household firearm access and homicide and suicide. We audited the letters to the editor emanating from those 16 articles. Each letter was coded for themes by two raters and analyzed using descriptive statistics and cluster analysis. For comparison, we also coded and analyzed the content of letters to the editor written in response to all other articles that were published in the same journal volumes where the firearm articles appeared.

**Results:**

We identified 30 letters regarding the gun in the home studies: 24 (80%) letters to the editor and 6 (20%) replies from original authors. Of the 24 letters to the editor, 30% contained no scientific discussion, 46% made a political reference, 17% criticized the original author’s character, and 25% criticized the journal. Moreover, 29% made a pro-gun reference, 25% made an anti-gun reference, 13% referred to the constitutional right to bear arms, 13% referred to the National Rifle Association (NRA), and 0% referred to advocacy organizations known to be in opposition to the NRA. Of these themes mentioned in letters to the editor, only the NRA was mentioned in a response by an original author. The median number of scientific citations in letters to the editor was one versus four in replies from original authors. In the articles on topics other than firearms that were analyzed as a point of comparison, only 8% contained no scientific discussion, 4% made a political reference, 2% criticized the authors’ character, and 0% criticized the journal.

**Conclusions:**

Letters to the editor in response to epidemiologic research on guns in the home contain considerable content that minimally advances scientific discussion; author responses meet a higher standard for science and civility, as do letters to the editor regarding research topics other than firearms. The scientific study of firearm violence could be better served with more letters containing greater scientific commentary and dissent.

## Review

Letters to the editor are an important venue for scientific discussion and for ensuring accountability of authors and journals (Tierney et al. 2015; Collier 2014; Slavov 2015). A number of studies set in different fields of research have analyzed letters to the editor for their content as a way to investigate different aspects of the roles that letters play and the ways in which letters are used. Horton, while serving as the editor of *The Lancet*, studied letters to the editor published in response to three randomized trials about hypertension that were reported in *The Lancet* (Horton 2002). His goal was to determine the extent to which recommendations stated in the letters to the editor were incorporated into practice guidelines published later. A primary purpose of that paper was communicate that letters to the editor serve the critical function of pointing out important weaknesses of published trials, and that the recommendations in those letters to the editor should be accounted for in future research and incorporated into practice guidelines that are based on that research. Letters to the editor are a potentially critical by-product of primary research that has the potential to advance the state of science.

Another example of research into letters to the editor is the study by Gøtzsche et al., who studied the adequacy of authors’ replies to criticism raised in letters to the editor (Gøtzsche et al. 2010). Gøtzsche et al. identified letters to the editor published in the *British Medical Journal* in response to research papers that generated substantive criticism. They coded the content in each letter to the editor by classifying the severity of each criticism (minor, moderate, major). They then determined how often the authors of the original research replied. Gøtzsche et al. found no relation between the severity of the criticism and the adequacy of the author's reply, and concluded that editors should ensure that authors take relevant criticism seriously and respond adequately to it. When viewed alongside the paper by Horton, it further argues that letters to the editor can communicate valuable information that has the potential to advance science.

We are particularly interested in letters to the editor regarding primary research conducted in topics that may be unusually contentious, in which both the public and scholars may have implacable, pre-existing conclusions that were formed prior to scientific study. Firearm violence is one such topic (Branas et al. 2009). Letters to the editor published in response to research on the health implications of keeping a gun in the home are potentially an excellent way to gauge and learn from this contentiousness.

As an example, one original study of guns in the home and suicide published in the *New England Journal of Medicine* (Kellermann et al. 1992) generated a letter to the editor that stated the following (Frey 1992):

To the Editor: …As for me, I have excellent health insurance, nursing home insurance, and insurance for home health care, but my ultimate insurance is my .357 Magnum.

This statement does not point out limitations of the study that might have led the authors to draw the conclusions they did, nor does it offer insight into how the research findings could meaningfully be incorporated to inform practice guidelines or public health policies. Indeed, this statement is terse, unscientific, and carries little if any potential to improve our understanding of health implications of firearm access, relative to the study on which it was commenting.

Firearm violence is challenging to study and has been profoundly under-resourced for decades compared with other biomedical and public health issues (Branas et al. 2005). Motivated by the possibility to better understand and advance research, discussion, and productive scientific dissent into firearm violence as a major societal concern, we conducted a multi-decade analysis of letters to the editor published in response to research on firearms in the home.

## Methods

The first author (DW) became interested in the topic investigated here when, in 2003, he published a study of guns in the home and homicide and suicide (Wiebe 2003), and had the opportunity to reply to a letter to the editor (Fritz 2004). A few prominent studies – with notable letters to the editor – had preceded it, and additional studies followed. Conveniently for our purposes, this body of epidemiologic studies investigating household firearm access as a risk factor for homicide and suicide was identified in a recent meta-analysis (Anglemyer et al. 2014). The meta-analysis, conducted by Anglemyer, Hovarth and Rutherford and published in *Annals of Internal Medicine* in 2014, was conducted by searching PubMed, EMBASE, the Cochrane Central Register of Controlled Trials, and Web of Science in August 2013. The authors selected all study types that assessed outcomes between participants with and without household firearm access. There were no restrictions on age, sex, or country. Based on their research results, the authors included in their meta-analysis 16 published articles reporting case-control studies or cohort studies of homicide or suicide that investigated the mortality risk associated with having a firearm in the home or not having access to a firearm. The authors of the meta-analysis concluded that the pooled odds ratio for suicide associated with access to firearms was 3.2 (95% CI, 2.4 to 4.4) and the pooled odds ratio for homicide associated with access to firearms was 2.0 (95% CI, 1.6 to 3.0).

In June 2016, we identified all letters to the editor that had been published in response to the 16 articles studied in the meta-analysis. We also identified replies to those letters that were written by the original study authors. First, we conducted a content analysis of these letters that emanated from the 16 articles, treating these letters as a case series and reporting on the themes and salient points that were communicated. We followed the methods for content analysis described by Zhang and Wild (Zhang & Wildemuth 2009). Specifically, by initially briefly reviewing the letters, we decided to use both terms (e.g., National Rifle Association) and themes (e.g., criticism of the author) as units of analysis. Then, having no prior work on this firearm topic to use as a starting point to follow, we used an inductive approach to develop a coding scheme to document the themes and content that we would code for in the letters. For example, regarding themes, we coded each letter as to whether it expressed scientific discussion, a political reference, a criticism of the original author, or a criticism of the journal editor. This was accomplished by reading each letter to identify the expression of each of these notions or ideas (Minichiello et al. 1990). Following conventional practice, portions of sentences or terms could be multiply coded as expressing more than one theme. As a general principle, we worked to develop a coding scheme that identified themes that were unique (i.e., internally homogenous, externally heterogeneous) (Lincoln & Guba 1985) and that addressed the range of themes that emerged that were relevant to our aim to understand notions being communicated in this focused universe of discourse. As part of this process, we developed a coding manual, consisting of theme names, rules for assigning codes, and examples, that evolved during the process. We then tested the coding scheme on a sample of text from several articles. Finally, two raters separately coded each letter to the editor according to whether any of 12 themes were expressed (yes/no). The coding was rechecked for consistency, and the results were discussed as a group to identify and discuss instances of disagreement to make final determinations.

Next, we used descriptive statistics and a cluster analysis to identify whether letters to the editor differed in content and tone from the letters written by original authors in their replies. The cluster analysis involved computing a matrix of Jaccard measures of similarity among the variables representing the 12 criteria used for coding, clustering the variables with an average linkage function, and plotting the results in a dendrogram. Stata Version 13 was used for analysis (StataCorp 2013).

In addition, we collected a separate set of letters to serve as a point of comparison. Specifically, we searched for and obtained each letter to the editor that was published in response to all other articles, regardless of topic and with no exclusions, that were published in the same journal volumes that contained the 16 firearm access articles that are our primary focus. We used the same coding scheme and methods described above to review and analyze the content of these letters.

## Results

Letters to the editor were published in response to eight of the 16 articles reporting findings of epidemiologic studies investigating firearms in the home as a risk factor for homicide or suicide. We identified a total of 30 letters, consisting of 24 (80%) letters to the editor and 6 (20%) that were written by an author of one of the articles to reply to a letter to the editor. Among the articles for which at least one letter to the editor was published, the number of letters to the editor that were published about a given article ranged from 1 to 8 (not including replies from the authors of the original articles). A letter containing the reply from the original author was published for five (63%) of the eight studies for which at least one letter to the editor was published.

Of the 24 letters to the editor regarding gun in the home articles, most were listed under a general heading (e.g., “Letters”) but six (25%) contained a descriptive title. For example, a title of one letter was “Flaws in study of firearm possession and risk for assault” (Wintemute 2010). The authors’ response was titled “Branas et al. response” (Branas et al. 2010). We know, given our involvement with that response, that the authors’ request to provide their own descriptive title for their reply was denied by the editor of the journal. Also as an example, a title of another letter to the editor was “Lies, damned lies, and statistics….” (Fritz 2004). The author’s reply was titled “In reply” (Wiebe 2004). Other letters to the editor were titled so that their content was explicit, for example “Bias when using dead controls to study handguns purchase as a risk factor for violent death” (Wiebe & Branas 2003). In this case, there was no author reply. While the aforementioned descriptive titles captured either the content or tone of the letter, the remaining three descriptive titles acted more as a shorthand of the original article’s title, presumably for ease of reference: “Firearm access and suicide” (Branas et al. 2009), “Guns and adolescent suicide” (Rosenberg et al. 1991), and “Gun availability and violent death” (Morgenstern 1997).

As noted above, we coded each letter and each reply in terms of whether they contained each of a number of themes of interest. Examples that met the criteria of the themes are as follows. Each of these examples is drawn from a letter to the editor rather than a letter from an author in reply.

- Scientific discussion: “Is the validity of data from an acquaintance or relative living apart from the case subject equivalent to the validity of data from a member of the same household?”
- Political reference: “Whether it is worth the social cost of stricter policies due to the small effect size, it should not be discounted (regarding firearm restriction policies).”
- Character critique: “Authors deliberately biased the study to a predictable gun control conclusion, therefore the results of the study are compromised.”
- Journal critique: “And, perhaps worse, the study calls attention to serious weaknesses in peer review by the *Journal*, funding by the Centers for Disease Control and Prevention, and the Public Health Service’s Office of Research Integrity, since it is riddled with methodological and research flaws caused by open anti-gun bias.”
- Pro-gun reference: “As for me I have excellent health insurance, nursing home insurance, and insurance for home health care, but my ultimate insurance is my .357 Magnum.”
- Gun control reference: “Restrictions to firearms should include rifles and shotguns, as shown by the study’s results.”
- National Rifle Association reference: “Clearly you have focused on the NRA’s lobbying and not on the fact that the NRA promotes responsible, safe handling of firearms for appropriate activities such as hunting, collecting, competitive shooting…and using a gun as a last-resort means of personal defense.”
- Constitution or 2nd Amendment reference: “The true issue, as always, is our willingness to accept the risks of human nature in return for the benefits of autonomy, self-reliance, and our constitutional rights.”
- Disagree with article: “The gun owners have characteristics, other than gun ownership itself, that increase their risk of suicide. Since these traits were not controlled for, it is impossible to say whether the availability of guns itself made any contribution to the suicides studied.”

Table 1 reports characteristics of the 24 letters to the editor and 6 letters written by authors in response. Of the 24 letters to the editor, 70% contained scientific discussion whereas the remaining 30% contained no scientific discussion. Also, 46% of the 24 letters made a political reference, 17% criticized the character of the original author, and 25% criticized the journal or editor for publishing the original article. Moreover, 29% made a pro-gun reference, 25% made a gun control reference, 13% referred to the National Rifle Association (NRA), and 0% referred to advocacy organizations known to be in opposition to the NRA, and 13% referred to the constitutional right to bear arms. The only one of these many topics that was referred to in the six letters written as replies by the original authors was the NRA. The median number of scientific references cited in letters to the editor was one, whereas in replies from original authors, the median number of scientific references cited was four.

**Table 1:** Characteristics of letters to the editors regarding gun in the home articles, letters written by authors of the original articles in response, and letters to the editors regarding articles on other topics

	Gun in the home articles		Other articles
	Letters to the editor *n* = 24	Author’s replies *n* = 6	Letters to the editor *n* = 57
	n (%)	n (%)	n (%)
Scientific discussion	17 (70.1%)	6 (100%)	52 (91.2%)
Political reference	11 (45.8%)	0 (0%)	2 (3.5%)
Character critique	4 (16.7%)	0 (0%)	1 (1.8%)
Journal critique	6 (25.0%)	0 (0%)	2 (3.5%)
Pro-gun reference	7 (29.2%)	0 (0%)	0 (0%)
Anti-gun reference	6 (25.0%)	0 (0%)	0 (0%)
National Rifle Association (NRA) reference	3 (12.5%)	2 (33.3%)	0 (0%)
Reference to organization in opposition to NRA	0 (0%)	0 (0%)	0 (0%)
Constitution or 2nd Amendment reference	3 (12.5%)	0 (0%)	0 (0%)
Disagree with article	17 (70.8%)	0 (0%)	32 (56.1%)
Number of scientific citations, median	1	4	4

**Fig. 1:**
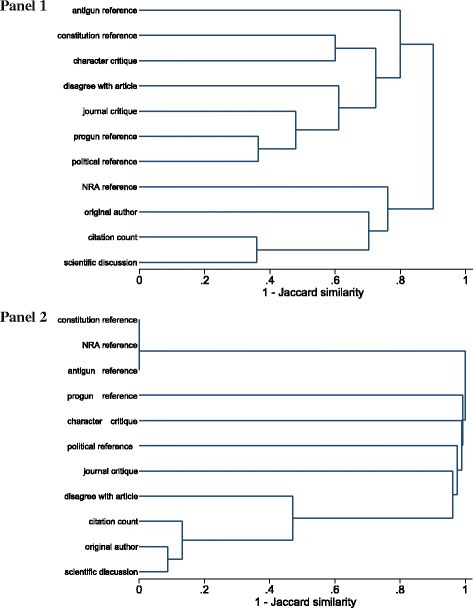
Cluster analysis dendrogram representing the co-occurrence of journal correspondence characteristics in letters to the editor regarding gun in the home articles (*Panel 1*) and articles on topics other than firearms (*Panel 2*). Note: The length of the bars, measured along the horizontal axis, connecting the themes represents the association among the themes, with shorter bars corresponding to greater similarity among clustered themes.

Results of the cluster analysis of letters to the editor in response to firearm access articles are reported in Fig. 1 (*Panel 1*). Bar length measured along the horizontal axis represents the magnitude of association among clustered themes and shorter bars connecting two themes indicates greater co-occurrence of those themes. The dendrogram shows that two broad clusters emerged, as indicated by the two primary branches. In the larger cluster displayed in the upper two-thirds of the graph, letters making a political reference also were likely to make a pro-gun reference. These letters were also likely to critique the journal and to convey disagreement with the original article. Also, articles making a reference to the Constitution or 2nd Amendment were also likely to critique the character of the author of the parent article. These letters were typically not written by the author of the original article, as revealed by this variable appearing in the second cluster portrayed in the lower third of the Fig. 1 (*Panel 1*). This second cluster indicates that replies from original authors commonly contained scientific discussion and cited a higher number of scientific references to support the positions stated in the letter to the editor. These letters also disproportionately made a reference to the National Rifle Association.

A common critique of articles on suicide was that methods other than firearm (i.e., gunshot) were not included. A common critique of articles on homicide was that results were limited to homicides categorized as “criminal” and that mortality as an outcome was not common in instances where guns are used as protection. Though personal anecdotes were common in letters across outcomes (suicide and homicide), the sentiments of these anecdotes were qualitatively different. In letters to the editor regarding articles on suicide, individuals shared personal experiences with patients and/or family members that made the research conducted all the more relevant for them. In the letters regarding homicide, personal reactions were grounded more so in gun ownership. Authors of these letters seemed to take offense to generalizations of gun owners as unsafe.

We also considered whether the critiques and questions asked in letters to the editor were addressed in the letters that the authors of the original articles wrote in reply. We found that without exception, the critiques and questions were addressed in letters that authors wrote in reply. It is also interesting to point out that, as reported in Table 1, a considerable number of letters to the editor contained political statements and went so far as to critique the character of the authors of the original articles. None of the letters written in reply contained political statements or included a character critique.

A summary of the characteristics exhibited in the letters to the editor that we collected for a point of comparison is also reported in Table 1. The articles to which these letters were responding addressed a range of public health and medical topics, including zinc intake, breastfeeding, religion and medicine, and neuromuscular disease. None addressed another aspect of firearms and public health. Almost all (92%) contained scientific discussion, only 3.5% made a political reference, and only 1.8% critiqued the author’s character, and only 3.5% were critical of the journal. The median number of citations was four. Only 56.1% disagreed with the article, whereas noted above, 70.8% of the letters responding to firearm access articles disagreed with the article.

A dendrogram based on these data tied to studies of topics other than firearms is reported in Fig. 1 (*Panel 2*). These results indicate that letters in reply by original authors were more likely than letters to the editors – to which they were replying – to contain scientific discussion, and authors’ replies generally had a higher count of scientific citations than did letters to the editor. These characteristics formed one apparent cluster. Otherwise, the diagram indicates “chaining” rather than clustering. That is, a generally decaying pattern of each category peeling off from the previous, which reveals simply that very few in any instances occurred of critiquing the journal, making a political reference, and so on.

## Conclusions

We find that letters to the editor in response to epidemiologic research on firearm access contain considerable content that does not advance the state of science in this field. Also, we found evidence that the authors of the original research on this topic, when they wrote replies, offered statements that met a higher standard for scientific content and had less incivility in tone. Moreover, we found that letters to the editor that were written in response to other, non-firearm topics that were published in the same journal volumes as the firearm access articles met a higher scientific standard and were more civil than were the letters in response to the firearm access articles. We believe this is the first study to investigate this issue.

There are multiple important considerations. We reported that letters written by an author of a firearm research article to reply to a letter to the editor regarding their article typically had a generic title like “Author responds,” whereas several letters to the editor had novel titles that were descriptive and were overtly critical of the published research. We noted that in at least one instance, the authors’ request to list a descriptive title for their response was denied by the journal editor. We do not know how often this happened for the articles we studied here. Even so, even this single example is an inequity that we believe should not exist. It is also important to point out that while letters to the editor were published in response to eight of the 16 epidemiologic studies and author replies were published in response to only five of those eight articles, we cannot say that this is evidence of another inequity. It may be the case that all authors were given the opportunity to reply and three of the authors declined.

One aspect of the cluster analysis indicated that reply letters written by authors of the original firearm access studies were disproportionately likely to refer to the National Rifle Association, as compared to letters to the editor. This is accurate, but should be explained in case the reason is not readily apparent. As seen in Table 1, whereas almost an equal number of letters (three) as responses (two) referred to the NRA, these references are proportionally far more common in responses than letters – 33% versus 13% percent, respectively. Hence the reason that the dendrogram indicates that references to the NRA were more common in replies from original authors than in letters to the editor.

Our study has limitations. We analyzed only published letters and replies. These may not represent the submitted letters overall; thus there may be selection bias on the part of editors. Also, authors may have been given the opportunity to reply and chosen not to do so. Second, we conducted the data theme coding ourselves rather than assigning the task to coders naïve to the broader goal of the paper, or coders who do not conduct research on firearms. Perhaps, then, biases of our own influenced the coding results. We aimed, as we do in all research that we conduct, to identify possible sources of bias and minimize the possibly they will lead to erroneous results. We believe that we have conducted this study responsibly. Also, the gun in the home research that we investigated is only one among many areas of research on firearms in the field of public health and medicine. It would be worthwhile to investigate letters to the editor from other areas, such as gun laws and their relation to firearm mortality. The content and tone of letters from other areas may differ from what we reported here.

Although we did include one group of studies as a point of comparison, we have not investigated thoroughly whether research in other fields of medicine or science prompts letters to the editor that challenge authors and editors in a fashion and tone similar to what we found regarding the research on guns in the home. To be comparable enough to enable a fair comparison, consider this. Suggesting that keeping a gun in the home could possibly harm the gun owner or household members, rather that solely keep them safe, is not only antithetical to the reason why may people in the United States own guns, protection; the suggestion may be taken as a threat to one’s desire to own guns, or to manufactures’ ability to sell guns. So, this alternative hypothesis regarding firearms may sound not only improbable but threatening. Perhaps a fair comparison can be made by considering the landmark study by Doll and Hill that, in the *British Medical Journal* in 1950, tested the possibility that cigarette smoking posed a risk for lung cancer (Doll & Hill 1950). Indeed, smoking was common at the time, largely considered harmless, and fueled by a tobacco industry with a revenue stream in mind. That article prompted one letter, published the following month, by one Lennox Johnston (Johnston 1950). His letter expressed disagreement with the original authors, posed alterative possible explanations for the findings, cited other literature – articles of his own published in the *British Medical Journal* and *The Lancet* as evidence and, with civility, suggested his impression that Doll and Hill may be addicted to tobacco. There was no reply published by the original authors.

Although authors including Horton and Gøtzsche et al., as discussed above, have described the role that letters to the editor should play in advancing the state of scientific research, we have not seen this position communicated explicitly by journals in their instructions to authors. Instructions to authors are typically very complete in terms of their requirements for submitting a research manuscript. But instructions regarding letters to the editor are typically perfunctory, and give guidelines for practical issues like word count but not for issues of purpose, content, or tone. Such is the case also for the recommendations for publishing scholarly work in medical journals, which was published in 2014 from the International Committee of Medical Journal Editors (ICJME 2014). This 17-page document details recommendations for publishing research papers and does mention letters to the editor, but gives no more guidance than to say letters to the editor can be published to highlight matters of debate to readers (Collier 2014; Slavov 2015).

We understand that there is value to drawing attention to matters to debate. This can be done not only in letters to the editor, but also in comments sections and online venues. But it is also important to recognize that it takes time and energy for authors to reply to critiques, including those that are misguided and do not have the potential to advance the field. And for the medium of the letter to the editor in particular, authors of the original research are typically given an opportunity to reply, and may therefore feel under pressure to do so to defend themselves. It is reasonable to expect authors to defend their science; it is another matter for editors to publish letters that put the authors in the position to have to defend their character, or that amount to enabling bullying (Collier 2014). A reasonable next step would be to revise letter to the editor guidelines to reflect the principles outlined by Horton and Gøtzsche et al. as a way to raise awareness in the field and encourage editors to publish letters that have the potential to advance the field and that do not unnecessarily distract original authors from their own attempts to do so.

## Abbreviation

NRA: National Rifle Association
